# Practice effects vs. transfer effects in the Simon task

**DOI:** 10.1007/s00426-020-01386-1

**Published:** 2020-08-07

**Authors:** Stefania D’Ascenzo, Luisa Lugli, Roberto Nicoletti, Carlo Umiltà

**Affiliations:** 1 Department of Philosophy and Communication, University of Bologna, Via Azzo Gardino, 23, 40122 Bologna, Italy; 2 Department of General Psychology, University of Padua, Padova, Italy

## Abstract

The Simon effect refers to the fact that, even though stimulus position is task-irrelevant, responses to a task-relevant stimulus dimension are faster and more accurate when the stimulus and response spatially correspond than when they do not. Although the Simon effect is a very robust phenomenon, it is modulated by practice or transfer from previous tasks. Practice refers to the modulation of the Simon effect as a function of number of trials. Transfer refers to the modulation of the Simon effect as a function of preceding tasks. The aim of the present study is to disentangle the role of practice and transfer in modulating the Simon effect and to investigate whether such modulation can be extended to a different response modality. Three experiments were conducted, which included three sessions: the Baseline session, the Inducer session and the Diagnostic session. The task performed in the Baseline and the Diagnostic sessions were comprised of location-irrelevant trials (i.e., they were Simon tasks). The task performed in the Inducer session required performing location-relevant trials (i.e., it was a spatial compatibility task with a compatible or an incompatible stimulus–response mapping). In the first and third experiments, participants were required to respond manually in all sessions. In the second experiment, the task performed in the Inducer session required manual response, while in the Baseline and Diagnostic sessions the tasks required ocular response. Results showed a reduced-Diagnostic Simon effect after both compatible and incompatible mapping in the Inducer session, regardless of whether response modality was the same or different. These results support the notion that the practice effect prevails over the transfer effect.

## Introduction

Performance, in both speed and accuracy, is influenced by the spatial relations between stimulus (S) and response (R), even when these spatial relations are not relevant to the task (see, e.g., Hommel & Prinz, 1997). Strong evidence of the importance of S–R spatial relations arises from studies that make use of the Simon task (e.g., Simon & Rudell, 1967; Simon, 1990; for reviews, see Rubichi, Vu, Nicoletti & Proctor, 2006; Proctor & Vu, 2006), in which participants are required to respond to a non-spatial feature (e.g., color) of a stimulus presented to the left or to the right of fixation by executing a spatially defined response (e.g., pressing the left or right response key). Even though stimulus position is task irrelevant, performance is faster and more accurate when stimulus position and response position spatially correspond (i.e., corresponding condition) compared to when they do not correspond (i.e., non-corresponding condition). A widely accepted explanation of this effect is based on dual-route models, which posit the existence of two routes that link stimulus to response: a direct or automatic route and an indirect or controlled route (e.g., De Jong, Liang & Lauber, 1994; Kornblum, Hasbroucq & Osman, 1990).

These models hypothesize that, when a stimulus appears, a slow, controlled route activates the required response on the basis of task-defined associations (Short-Term Memory, STM, links), while, in parallel, a fast, automatic route activates the response that spatially corresponds to the stimulus location through pre-existing, task-independent S–R associations (Long-Term Memory, LTM, links). In corresponding trials, the automatic route and the controlled route activate the same response, leading to fast responses. In non-corresponding trials, the controlled route activates a response that is different from the one activated by the automatic route, producing a conflict that results in slower and less accurate responses. This view predicts that the magnitude of the Simon effect should depend on the relative strength of the activation reached by the two routes.

LTM links are rather pervasive and manifest themselves in disparate conditions. Thus, the Simon effect emerges with various stimulus modalities (visual: e.g., Proctor and Lu, 1994; Tagliabue, Zorzi & Umiltà, 2002; Wühr & Ansorge 2005; auditory: e.g., D’Ascenzo, Lugli, Baroni, Guidotti, Rubichi, Iani & Nicoletti, 2018; Simon & Rudell, 1967; Vu, Proctor & Urcuioli, 2003; tactile: e.g., Salzer, Aisenberg, Oron-Gilad & Henik, 2013) and with various response modalities (motor: e.g., Rubichi, et al., 2006; vocal: e.g., Wühr, 2006; oculo-motor: e.g., Lugli, Baroni, Nicoletti & Umiltà, 2016; Verghese, Mattingley, Palmer & Dux, 2017). Also, it can be observed with both horizontal and vertical stimulus–response arrangements (e.g., Umiltà, Rubichi & Nicoletti, 1999; Vu, Proctor & Pick, 2000; Vallesi, Mapelli, Schiff, Amodio & Umiltà, 2005; Weeks, Proctor & Beyak, 1995).

In spite of its robustness and pervasiveness, the emergence and consistency of the Simon effect can be modulated in experimental conditions that produce what, from now on, will be referred to here as practice effects (e.g., Proctor & Lu, 1999) and transfer effects (e.g., Tagliabue, Zorzi, Umiltà & Bassignani, 2000; Lugli, Iani, Milanese, Sebanz, & Rubichi, 2015). The term *practice effect* refers to the observation that the Simon effect decreases after the participant has performed the task for a number of trials (see Proctor & Lu, 1999, Experiment 1). Proctor and Lu demonstrated that the visual Simon effect ‘decreased from its initial value but persisted at a reduced magnitude for at least 1800 trials’ (p. 74). In their Experiment 1, participants performed the Simon task for 1800 trials, subdivided into three sessions. The authors found a Simon effect of 22 ms (ms) in the first session, which significantly decreased to 14 ms in the second and third sessions. Thus, the visual Simon effect was reduced but not eliminated after extensive practice, as already observed for the auditory Simon effect (Hommel, cited in Prinz, Aschersleben, Hommel & Vogt, 1995; Simon, Craft & Webster, 1973). Proctor and Lu suggested that the Simon effect decreases as a function of practice because the participant learns to ignore or to suppress the irrelevant locational information.

The term transfer effect refers to the modulation of the Simon effect as a function of a different, often preceding, task (see Proctor & Lu, 1999, experiments 2 and 3). The standard version of the transfer paradigm involves two sessions, the Inducer session and the Diagnostic session, which can be presented in a task-switching version (i.e., the trial of the two tasks performed in the two sessions are intermixed; e.g., Marble & Proctor, 2000), or in a sequential-task version (i.e., the task performed in the Inducer session precede the task performed in the Diagnostic session; e.g., Proctor & Lu, 1999, Experiments 2 and 3). Both versions require manual responses and comprise either location-relevant trials (i.e., a spatial compatibility task, with compatible or incompatible S–R pairings, as the task performed in the Inducer session) or location-irrelevant trials (i.e., a Simon task as the task performed in the Diagnostic session).

This standard transfer paradigm was previously employed in several studies, which have consistently found that, after the task performed in the Inducer session with a spatially incompatible S–R mapping (i.e., responding to the left stimulus with the right key and to the right stimulus with the left key), the Simon effect in the Diagnostic session was eliminated (Tagliabue, et al., 2000) or reversed (e.g., Proctor & Lu, 1999; Iani, Rubichi, Gherri & Nicoletti, 2009; Lugli, Iani, Nicoletti & Rubichi, 2013; Rubichi, Gherri, Nicoletti & Umiltà, 2005; Soetens, Maetens & Zeischka, 2010). A widely accepted account of the transfer effect found with this standard paradigm invokes the transfer of specific STM/LTM associations. It appears that, when a spatially incompatible S–R mapping is employed (i.e., the Inducer session), new (non-corresponding) associations of spatially incompatible S–R locations become active and are strengthened. These would become new LTM links. In the Simon task performed in the Diagnostic session, when stimulus location is irrelevant, the acquired associations (i.e., the newly acquired, spatially incompatible LTM links) remain active and the spatially incompatible response is automatically activated and competes with the current spatially compatible response. Also, the newly acquired incompatible LTM links compete with the previously existing compatible LTM links. If the new and old LTM links are of equivalent strength, the Simon effect is null. If the new incompatible LTM links prevail, a reverse Simon effect is observed. In contrast, when a spatially compatible S–R mapping is employed in the Inducer session, the already existing (corresponding) long-term S–R links are confirmed, or, perhaps, even strengthened. Thus, a regular (or increased) Simon effect is observed in the Diagnostic session (see, e.g., Iani, et al., 2009, experiment 2).

The standard transfer paradigm has been investigated also with some variations, such as different stimuli (e.g., Proctor, Yamaguchi, Zhang & Vu, 2009) and different response modalities in the two sessions (e.g., Treccani, Ronconi & Umiltà, 2017) or with three sessions, thus including a Baseline session (e.g., Wang & Weeks, 2014).

For what concerns stimulus modality, Proctor et al. (2009) examined whether, in the visual modality, transfer occurs for various combinations of physical-location, arrow-direction, and location-word in the practice and transfer sessions. They observed a transfer effect across stimuli that require visual-spatial codes (i.e., physical-location and arrow-direction), but little transfer effect for stimuli that require semantic-spatial codes (i.e., location-words).

Another variation of the standard transfer paradigms (i.e., manual key presses) which has been investigated consists in having different response modalities between Baseline/Diagnostic and Inducer sessions. The issue of what happens if response modality is changed between the two sessions (i.e., when the tasks performed in the Inducer and Diagnostic sessions require different response modalities) has attracted little interest. A first attempt in this direction was made by Yamaguchi, Chen & Proctor, (2015) and by Treccani, et al., (2017) with paradigms consisting in sequential and switching tasks, respectively. Yamaguchi, et al., (2015), in their Experiments 1 and 2, employed a vocal response and varied the type of stimuli (physical locations vs. spatial words) between the tasks performed in the Inducer and the Diagnostic sessions. In Experiments 3 and 4, instead, they varied the modality of the response (vocal or manual) between the tasks performed in the two sessions. They found that the magnitude of the Simon effect with vocal responses was more affected when stimulus modality in the tasks performed in the Inducer and Diagnostic sessions was the same than when it was different. In addition, they found that there was little influence of the Inducer session when the task has an incompatible mapping when response modality was different from the one of the task performed in the Diagnostic session. The authors concluded that the contextual overlap in stimuli and response modalities is an important determinant of the transfer effect (also, see Yamaguchi & Proctor, 2009). Treccani, et al., (2017) investigated the role of changing response mode (manual, vocal or pedal) between the tasks performed in the Inducer session and the Diagnostic session. They observed a modulation of the Simon effect when response mode was the same in the task of the Inducer and Diagnostic sessions (i.e., manual or pedal in either), whereas no transfer occurred when response mode was different (i.e., vocal and manual or pedal and manual). It should be noted that the authors discussed the possibility that, in their study, the reason why the transfer did not occur was attributable to a change in the response device rather than to a change in response mode.

Up to now, many studies investigated the role of the overlapping of stimulus features and response features in the transfer effect (e.g., Tagliabue, et al., 2002; Proctor, et al., 2009). For what concerns stimulus features, several works have demonstrated that it does not seem to be mandatory to have an overlap in stimulus features for a transfer effect to occur from an Inducer session to a Diagnostic session (e.g., Tagliabue, et al., 2002; Treccani, et al., 2010). That suggests the existence of a spatial remapping when different stimuli are at play in the tasks performed in the two sessions. In contrast, in the case of response features, a substantial overlap between response modalities seems necessary for the transfer effect to occur (e.g., Treccani, et al., 2017), as attested by the fact that no transfer effect was observed when different response modalities were employed in the tasks performed in the two sessions (e.g., Yamaguchi, et al., 2015; Treccani, et al., 2017). A different account has been proposed for those paradigms in which there is no feature overlap between the tasks in the Inducer and the Diagnostic sessions but the transfer effect occurs nonetheless. For example, Treccani, et al., (2010) employed a switching-tasks paradigm (similar to that used by Hedge & Marsh, 1975), in which the spatial correspondence effect in the task required in the Diagnostic session (i.e., shape discrimination) was obtained by combining it with the task required in the Inducer session (i.e., color discrimination), whose mapping was compatible or incompatible. The authors demonstrated that the logical transformation induced by an incompatible task transferred to the task in the Diagnostic session, independently of specific stimuli dimensions. That shows that a transfer effect can occur even when there is no feature overlap between the stimuli dimension of the task performed in the two sessions (e.g., colors and spatial dimensions, as in Hedge & Marsh, 1975). In the case of paradigms in which different stimuli dimension are employed, and there is no feature overlap between stimuli dimension or response modalities, the transfer effect can be explained as resulting from the transfer of a logical recoding rule, named also “respond-opposite” account (see also Treccani, et al., 2017).

For what concerns changes of the standard transfer paradigm with three sessions, few studies have employed such paradigm, which implies asking participants to perform a Simon task also before the Inducer session. This initial Simon task (to which from now on we will refer to as the Baseline session) is aimed at providing a baseline against which to assess the size of the Simon effect observed in the Diagnostic session, that is the Simon task that can be affected by the task performed in the Inducer session (Wang & Weeks, 2014; D’Ascenzo, Iani, Guidotti, Laeng & Rubichi, 2016; Verghese, et al., 2017). Wang & Weeks (2014) investigated how the acquisition of a new S–R mapping rule may alter the neural mechanisms of the cognitive control system by employing fMRI with a paradigm that had a Baseline session (Simon task), an Inducer session (i.e., compatible and incompatible mapping) and a Diagnostic session (Simon task). D’Ascenzo, et al. (2016) investigated the relation between sequential modulation and transfer effect in the Simon task by means of pupil dilation and reaction time. They implemented a three-session paradigm in which a Simon task was performed in the Baseline session, followed by a task with an incompatible mapping in the Inducer session, and then by a Simon task performed in the Diagnostic session. Important for our present aims is the study by Verghese, et al. (2017), who employed pro-saccades and anti-saccades in the Inducer sessions. In their spatial compatibility task, saccadic responses, instead of manual responses, were performed to test transfer effects in a manual Simon task. They used an integrated Simon-Stroop task, that is, a paradigm that belongs to Type 8 ensembles in Kornblum, Stevens, Whipple & Requin (1999)’s taxonomy. In it there was a dimensional overlap, not only between the irrelevant stimulus position and the response positions (which would make a Type 3 ensembles, as in a typical Simon task), but also between both relevant and irrelevant stimulus features. It is worth noting that, in the standard pro-saccade–anti-saccade task (e.g., Hallett, 1978), participants are required to direct their gaze, as fast and as accurately as possible, toward a sudden onset stimulus or away from it. Results show slower latencies of anti-saccades than of pro-saccades. Verghese, et al. (2017) observed a significant reduction of the Simon effect in the Diagnostic session compared to the Simon effect in the Baseline session after the anti-saccade task in the Inducer session. In contrast, Simon effect in the Diagnostic session was not affected after the tasks in the Inducer session consisting in performing either a simple fixation or pro-saccades. The authors maintained that new associations, such as a “respond-opposite” rule (Vu, 2007), learned in the anti-saccade task of the Inducer session, can be transferred to a different session, resulting in a reduced Simon effect in the Diagnostic session, even though the two tasks (i.e., pro-saccade–anti-saccade and the Simon tasks) require different processes.

It should be noted that previous studies (e.g., Iani, et al., 2009; Soetens, et al., 2010), which had explored the transfer effect with the standard paradigm (i.e., two sessions and an incompatible task in the Inducer session) had reported null or even reverse Simon effects in the Diagnostic session. In contrast, Verghese, et al., (2017), who made use of a Baseline session, reported a reduced but still significant Simon effect in the Diagnostic session after an incompatible task in the Inducer session. Thus, it is reasonable to ask whether the results obtained by Verghese, et al., (2017) depended on a transfer effect across different response modalities or rather on a practice effect, like the one observed by Proctor & Lu, (1999), brought about by performing the Baseline session first and then the Diagnostic session. To clarify this issue, one needs to perform an experiment similar to those reported by Verghese, et al., (2017). That is, there needs to be compatible task in the Inducer session, not only incompatible task. The evidence will support an effect of practice only if the magnitude of the Simon effect decreases regardless of whether the task in the Inducer session is compatible or incompatible. If the Simon effect decreases only when the task in the Inducer session is incompatible, then that would be evidence in favor of a transfer effect. This issue will be investigated in the current study.

Besides trying to discriminate between transfer effects and practice effects, we explored whether the effects of LTM links acquired during the task performed in the Inducer session are specific to the response modality employed in that task. We did so by employing a type of response that had not been investigated before, that is ocular responses. In general, the ocular Simon effect (i.e., a Simon effect observed in a Simon task with saccadic responses) has seldom been investigated (for exceptions, see Buetti & Kerzel, 2010; Khalid & Ansorge, 2013). Recently, Lugli, et al. (2016) described the ocular Simon effect as depending on cognitive interference arising within the conditional route, in which the relevant stimulus feature is translated into a response. In contrast, the manual Simon effect appeared to be due to specific mechanisms of visuomotor links, which associates it with the automatic activation of the corresponding response through the unconditional route (i.e., Wascher, Schatz, Kuder & Verleger, 2001). Consequently, different modulations of the ocular and manual Simon effect in the Diagnostic session can be expected in case of a transfer effect.

Not surprisingly, studies concerning transfer effects involving ocular responses are even fewer. As already noted, the first attempt to address this issue was reported in the study of Verghese, et al. (2017). These authors employed an ocular task in the Inducer session to test transfer effects to a manual task in the Diagnostic session. However, to date no one has investigated the inverse condition, that is, the influence of a manual task in the Inducer session on a task that requires ocular responses in the Diagnostic session.

In the present study we employed a sequential-tasks paradigm, as Verghese, et al. (2017) did, with three sessions, the Baseline session (a version of the Simon task), the Inducer session, and the Diagnostic session (the same version of the Simon task as that performed in the Baseline session). As stated above, we investigated whether a reduction of the Simon effect in the Diagnostic session induced by performing the Simon task twice (Baseline session and Diagnostic session) is attributable to a practice effect or to a transfer effect. To this aim we employed a three-session version of the standard transfer paradigm, which included a Baseline session that usually is missing in the two-session version.

We would like to stress that the distinction between practice effect and transfer effect is critical to the present study. With the former term we refer to the basic fact that RTs become faster and more accurate after the participants have performed several trials in which spatially defined stimuli are responded to with spatially defined responses. Whether the link between stimulus and response is corresponding or non-corresponding is irrelevant. Our idea is that the practice effect originates because subjects practice forming spatial codes for both stimuli and responses and also practice connecting these spatial codes. The fact that trials are corresponding or non-corresponding is immaterial because in either case spatial codes are formed for both stimulus and responses and the two spatial codes are then connected. However, the link between the spatial stimulus code and the spatial response code is less direct in the case of non-corresponding trials and responses are slower. As a consequence, non-corresponding trials benefit more from practice and the Simon effect decreases with practice. For transfer effect, we refer to the observation that the rule that links stimuli to responses in a task (i.e., the Inducer session) is transferred to a different, often subsequent, task (i.e., the Diagnostic session). Therefore, for a transfer effect to occur what matters is learning a rule for linking stimuli to responses, whereas, as said, for a practice effect to occur what matters is making use of spatial stimulus codes and of spatial response codes. The prediction is that, in the case of practice, the Simon effect in the Diagnostic session should decrease after the task performed in the Inducer session, regardless of whether it is compatible or incompatible. In contrast, in the case of transfer, the Simon effect in the Diagnostic session should become null, or invert, only after an incompatible task performed in the Inducer session.

Three experiments were conducted. In the first experiment, we made use of tasks that all employed the manual response modality. In the second experiment, we made use of the same tasks in a condition of cross response modality (i.e., manual and ocular). In the third experiment, we made use of tasks that employed the same response modality (i.e., manual) and the same stimuli across the three sessions. The first two experiments started with a Baseline session, which was identical to the subsequent Diagnostic session. In between the two sessions, two groups of participants were asked to perform a manual task with compatible or incompatible mapping in the Inducer session (between participants’ condition). In the first experiment, the Baseline and the Diagnostic sessions consisted both in a manual Simon task, while in the second experiment the Baseline and the Diagnostic sessions were ocular Simon tasks. The third experiment was a replication of the first experiment (i.e., manual response modality) except that only a group of participants was tested. They were asked to perform a task with incompatible mapping in the Inducer session, and in all three sessions identical stimuli were employed, that is, black circles and black triangles.

For the first two experiments, we investigate, by means of a three-way interaction, the relation between the Mapping of the task performed in the Inducer session (compatible vs. incompatible), the Session (Baseline vs. Diagnostic) and Correspondence (corresponding vs. non-corresponding).

We hypothesized that, if the practice effect prevailed over the transfer effect, the three-way interaction would be not significant in either experiment, showing that the two types of mapping performed in the Inducer session, i.e., compatible or incompatible, had caused a reduction of the Simon effect in the Diagnostic session, which, however, in accord with what had been found by Proctor and Lu (1999), remained present and significant. In other words, the absence of significant three-way interactions would corroborate the notion that practice in the Baseline and Inducer sessions had affected the Diagnostic session in the same way. More specifically, it would appear that, regardless of the nature, compatible or incompatible, of the mapping and regardless of the nature, same or different, of response modality, the task performed in the Inducer session had produced the same practice effect.

In contrast, if the transfer effect prevailed, we expected to find a significant three-way interaction in either experiment. This significant interaction would result from a regular or increased Simon effect in the Diagnostic session when the task performed in the Inducer session has a compatible mapping and from a null or reversed Simon effect in the Diagnostic session when the task performed in the Inducer session has an incompatible mapping. Remember that the latter was the outcome of previous studies that lacked a Baseline session and employed only the Inducer and the Diagnostic sessions (e.g., Iani, et al., 2009).

Considering response modality, we predicted that, if the match of response modality between the tasks performed in the Inducer and the Diagnostic sessions is critical, we should have observed a modulation of the Simon effect in the Diagnostic session only in the first experiment, that is in the experiment with an incompatible mapping in the Inducer session, as already observed in previous studies (e.g., D’Ascenzo, et al., 2016; Wang & Weeks, 2014). In the second experiment no modulation of the ocular Simon effect in the Diagnostic session should emerge (in accord with what was found by Treccani, et al., 2017; who did not employ a Baseline session, though). If the match of response modality between the two tasks employed in the two sessions is not critical, then also in the second experiment a modulation of the ocular Simon effect in the Diagnostic session should emerge, depending on the mapping performed in the task of the Inducer session.

In the third experiment we expected to confirm the decrease of the manual Simon effect in the Diagnostic session after a manual task performed in the Inducer session. This time, however, that result would be obtained by employing stimuli that had the same color as those used in the Baseline and Diagnostic sessions. This experiment was performed, by following the suggestion of an anonymous reviewer, to rule out the possibility that the results observed in the first experiment depended on the fact that the stimuli used in the task performed in the Baseline and the Diagnostic sessions (i.e., blue and red squares) were too dissimilar from those used in the task executed in the Inducer session (i.e., black squares).

## Experiment 1

### Method

#### Participants

Fifty-eight^1^ students of the University of Bologna (38 female, 57 right-handed, *M*_age_ = 21, SD_age_ = 2.5) served as participants and received course credits for participation. All reported to have normal or corrected-to-normal vision and were naïve as to the purpose of the experiment.

#### Apparatus, stimuli and procedure

The experiment was conducted in a quiet room, where the light was dimmed. Stimuli were presented on a Dell 22 inch (56 cm) video monitor (refresh rate: 60 Hz; resolution: 1680 × 1050 pixels) on a white background. The viewing distance was 60 cm. Stimuli presentation and response collections were controlled by the Experiment CenterTM software (version 3.2) for both Simon task sessions and E-Prime Professional v2.0 software (https://www.pstnet.com) for the Inducer task.

Stimuli^2^ were black, red, or blue squares (3.05° × 3.05°) that appeared at the center of two dotted rectangles (5.05° × 6.5°) presented to the left or to the right of a fixation cross. The border of each stimulus and that of the dotted rectangles were 1.7° apart. The border of each rectangle and that of the fixation cross were 6.2° apart.

Participants were required to perform first a manual Simon task (i.e., Baseline session), second a spatial compatibility manual task with a compatible (*N* = 30 ss) or incompatible (*N* = 28 ss) S–R mapping (i.e., Inducer session) and third a manual Simon task (i.e., Diagnostic session).

**Fig. 1:**
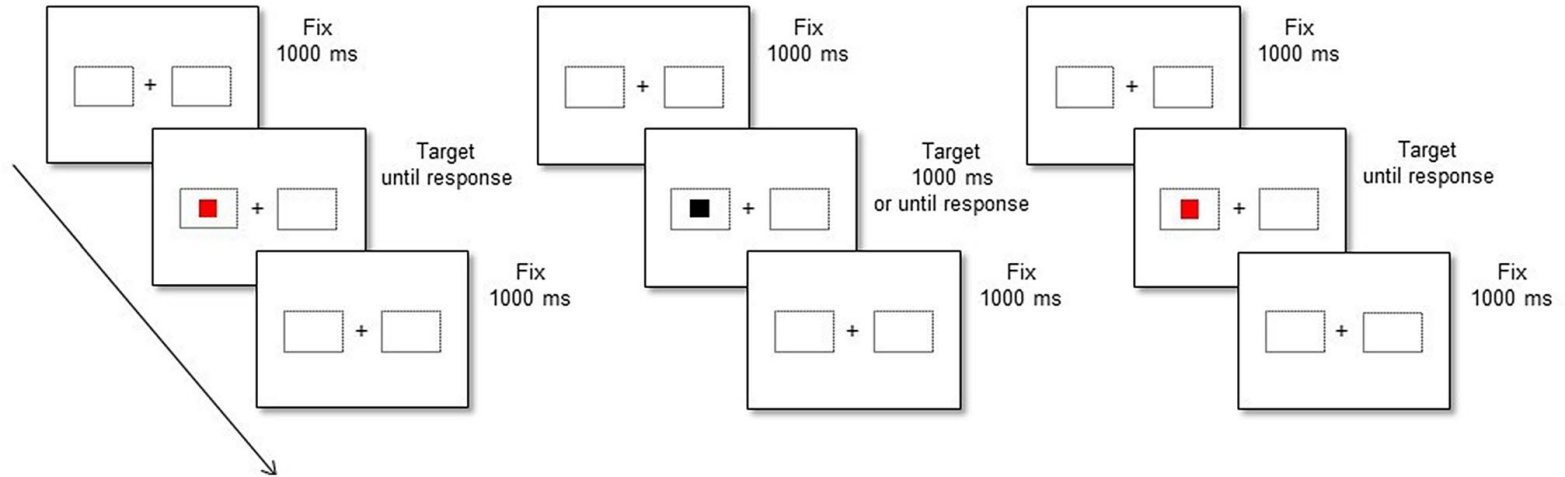
Temporal sequence of a representative trial in the manual Simon task performed in the Baseline (leftmost panel) and in the Diagnostic sessions (rightmost panel), and in the spatial compatibility manual task performed in the Inducer session (middle panel). Note that stimuli are not drawn to scale. See the electornic version for colored figure.

In the Inducer session, stimuli were black squares that appeared at the center of two dotted rectangles presented to the left or to the right of a fixation cross. Their size was the same as that of the target stimuli. In the compatible condition, participants were asked to respond as quickly and accurately as possible to the left stimulus by pressing the left response key and to the right stimulus by pressing the right response key (the “Ctrl” and the “Alt” keys on the QWERTY keyboard, respectively). In the incompatible condition, participants were asked to respond as quickly and accurately as possible to the left stimulus by pressing the right response key and to the right stimulus by pressing the left response key. A trial started with the fixation cross which was presented at the center of the screen for 1000 ms. Subsequently, the stimulus appeared and remained present for 1000 ms or until a response was made. The trial terminated if the participant did not respond within 1000 ms (see Fig. 1, middle panel). The Inducer session consisted of 144 trials that were divided into three blocks of 48 trials each.

In the Baseline and Diagnostic sessions, stimuli were red and blue squares. Half of the participants were instructed to respond to the blue stimulus by pressing the left response key and to the red stimulus by pressing the right response key (the “Alt” or the “Ctrl” keys on the QWERTY keyboard, respectively). The other half experienced the opposite mapping rule. At the beginning of the experiment, the fixation cross and the dotted rectangles appeared on the screen and remained visible throughout the whole experiment. Participants were required to fixate the cross for 1000 ms, and then the stimulus appeared and remained there until the participant’s response (see Fig. 1, left and right panel for Baseline and Diagnostic sessions, respectively). For both sessions, the Simon task consisted of 192 trials that were divided into four blocks of 48 trials each, preceded by 20 practice trials or 8 practice trials (in the Baseline or in the Diagnostic session, respectively).

### Analysis

Reaction times (RTs) that were 2 SD faster or slower than each participant’s mean (4.03% and 3.3% of the total trials for the Baseline and the Diagnostic sessions, respectively) and errors (4.7% and 4.7% of the total trials for the Baseline and the Diagnostic sessions, respectively) were excluded from the analysis on RTs.

The Simon effect, in terms of RTs, for both the Baseline and the Diagnostic sessions, was computed by comparing the RT to corresponding trial (i.e., the position of the response corresponded to the position of the stimulus) and the RT to non-corresponding trial (i.e., the position of the response did not correspond to the position of the stimulus).

For each experiment, two analyses will be reported. The first includes all participants, while the second includes participants that showed a positive Simon effect (i.e., non-corresponding RT > corresponding RT; see also Verghese, et al., 2017) in the Baseline session.^3^ Because the aim of the study was to investigate the role of practice and transfer effects on a Simon effect in the Diagnostic session by comparing it with a Simon effect in the Baseline session, it was deemed to be of paramount importance that participants reported a positive Simon effect in the Baseline session. This second analysis was rendered even more necessary by the fact that Verghese, et al. (2017) had excluded participants who did not show a positive Simon effect, in terms of RTs, in the Baseline session. Because we aimed at comparing our results with those of Verghese et al., we had planned from the outset to take into consideration only those participants that started with a positive Simon effect. Four participants did not meet the criterion of manifesting a positive Simon effect in the Baseline session and consequently were excluded from the second analysis, which was thus performed on 54 (35 females, all right-handed i_age_ = 21, SD_age_ = 2.1) out of 58 participants.

Two repeated measures ANOVAs were conducted on both RTs and arcsine-transformed error rates (ERs, to approximate a normal distribution; Winer, 1971) with Mapping (Compatible vs. Incompatible) as between-subject factor and Session (Baseline vs. Diagnostic) and Correspondence (Corresponding vs. Non-corresponding), as within-subjects’ factors for both samples. Huynh–Feldt correction was used when appropriate. The effect size was estimated by calculating the partial eta squared statistic (*η*^2^_p_).

## Results

*RTs* The first analysis on RT on the entire sample (*N* = 58) showed significant a main effects of Session *F*_(1, 56)_ = 22.490, MSE = 23,775.184, *p* < 0.001, *η*_*p*_^2^ = 0.287 and Correspondence *F*_(1, 56)_ = 81.534, MSE = 18,573.364, *p* < 0.001, *η*_*p*_^2^ = 0.593. Responses were faster in the Diagnostic session (409 ms) than in the Baseline session (429 ms), and in Corresponding (410 ms) than Non-corresponding trials (428 ms).

**Fig. 2:**
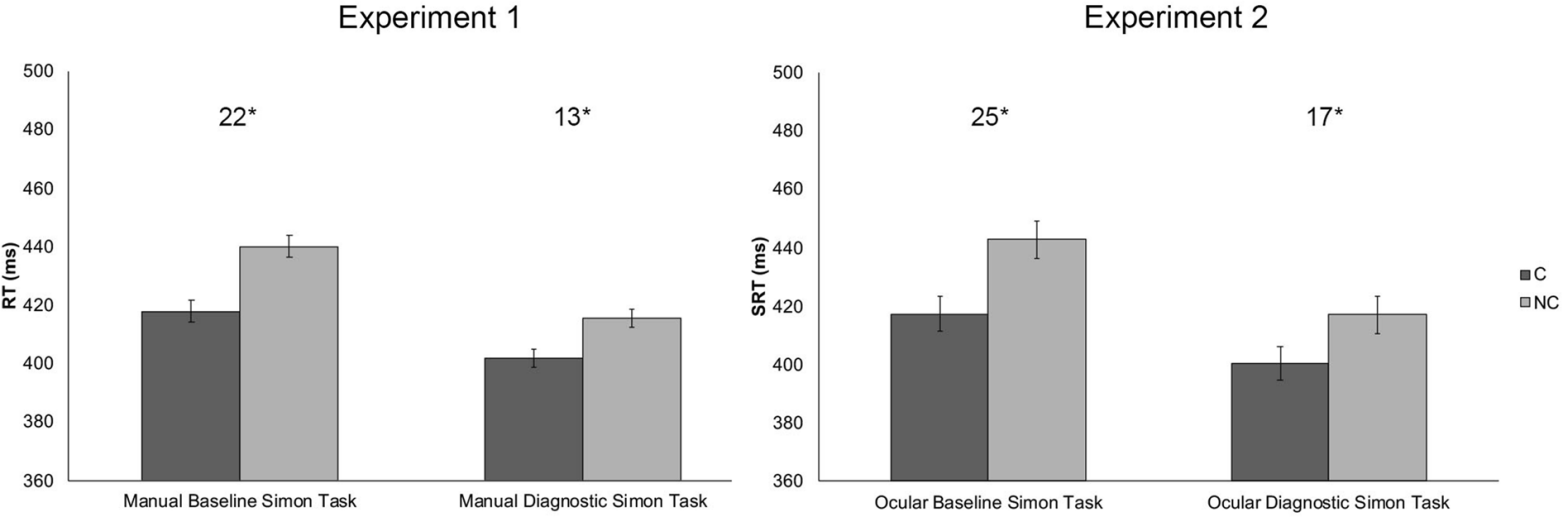
Mean reaction time (RT; ms) for Correspondence as a function of Sessions in Experiment 1 (left panel) and mean saccadic reaction time (SRT; ms) for Correspondence as a function of Sessions in Experiment 2 (right panel). Error bars indicate standard errors of the mean adjusted for within participants design (Loftus & Masson, 1994). The magnitude of the Simon effect, for the separate conditions, is reported on top. Asterisks denote significant values (*p < 0.005). *C* corresponding, *NC* non-corresponding

The interaction Session × Correspondence was significant *F*_(1, 56)_ = 9.385, *p* < 0.005, *η*_*p*_^2^ = 0.14 indicating that the Simon effect differed between the two sessions, regardless of the Mapping: 22 ms in the Baseline session and 13 ms in the Diagnostic session. Both effects were significant as shown by paired sample *t* tests (*t*_(57)_ = 9.745, *p* < 0.001 and *t*_(57)_ = 5.530, *p* < 0.001, Baseline and Diagnostic sessions, respectively). See Fig. 2, leftmost panel.

Interestingly, the interaction Mapping × Session × Correspondence was not significant *F*_(1, 56)_ = 1.160, *p* = 0.286, indicating that no modulation occurred between the Simon tasks performed in the Baseline and Diagnostic sessions related to the Mapping of the Inducer session. No other main effect, nor interactions were significant (*F*_*s*_ < 1).

The second analysis on RTs, performed on the sample with only participants (*N* = 54) with a positive Simon effect in the Baseline session, showed significant a main effects of Session *F*_(1, 52)_ = 17.594, MSE = 17,357.302, *p* < 0.001, *η*_*p*_^2^ = 0.253 and Correspondence *F*_(1, 52)_ = 93.506, MSE = 19,834.024, *p* < 0.001, *η*_*p*_^2^ = 0.643. Responses were faster in the Diagnostic session (407 ms) than in the Baseline session (425 ms), and in Corresponding (407 ms) than Non-corresponding trials (426 ms).

The interaction Session × Correspondence was significant *F*_(1, 52)_ = 14.190, *p *< 0.001, *η*_*p*_^2^ = 0.21 indicating again that the Simon effect differed between the two sessions, regardless of the Mapping: 24 ms in the Baseline session and 14 ms in the Diagnostic session. Both effects were significant as shown by paired sample *t* tests (*t*_(53)_ = 11.188, *p* < 0.001 and *t*_(53)_ = 5.532, *p* < 0.001, Baseline and Diagnostic sessions, respectively).

Interestingly, the interaction Mapping × Session × Correspondence was not significant *F*_(1, 52)_ = 1.190, *p* = 0.280. No other main effect, nor interactions were significant (*F*_*s*_ < 1).

*ERs* The first analysis on ER on the entire sample (N = 58) showed a significant main effect of Correspondence *F*_(1, 56)_ = 22.188, MSE = 0.162, *p* < 0.001, *η*_*p*_^2^ = 0.284. Participants made more errors in Non-corresponding (5.8%) compared to Corresponding trials (3.7%). The interaction Session × Correspondence was significant *F*_(1, 56)_ = 11.124, MSE = 0.044, *p* = 0.002, *η*_*p*_^2^ = 0.166, indicating that participants, in both Baseline and Diagnostic sessions, made more errors in Non-corresponding (6.2% and 5.2%, respectively) compared to Corresponding trials (3.2% and 4.1%, respectively). The difference was significant in the Baseline session (*t*_(57)_ = 5.685, *p* < 0.001) and close to significance in the Diagnostic session (*t*_(57)_ = 1.888, *p* = 0.064). No other main effects, nor interaction were significant (*F*_*s*_ < 1).

The second analysis on ER on the sample including only participants with a positive initial Simon effect (*N* = 54) showed a significant main effect of Correspondence *F*_(1, 527)_ = 23.190, MSE = 0.165, *p* < 0.001, *η*_*p*_^2^ = 0.308. Participants made more errors in Non-corresponding (5.9%) compared to Corresponding trials (3.7%). The interaction Session × Correspondence was significant *F*_(1, 52)_ = 10.954, MSE = 0.045, *p* = 0.002, *η*_*p*_^2^ = 0.174, showing that participants, in both Baseline and Diagnostic sessions, made more errors in Non-corresponding (6.4% and 5.3%, respectively) compared to Corresponding trials (3.2% and 4.2%, respectively). The difference was significant in the Baseline session (*t*_(53)_ = 5.922, *p* < 0.001) and close to significance in the Diagnostic session (*t*_(53)_ = 1.818, *p* = 0.075). No other main effects, nor interaction were significant (*F*_*s*_ < 1).

### Bayesian analyses

Based on the procedure of null hypothesis significance testing (NHST; Wagenmakers, 2007), the null hypothesis can never be accepted. Therefore, to choose between the null and alternative hypotheses, we compared the data and performed a Bayesian hypothesis testing using the BIC approximation (Wagenmakers, 2007; Altoè, 2014). This analysis^4^ was aimed at comparing the relative plausibility of the null and the alternative hypotheses for the three-way interaction involving Mapping, Session and Correspondence, which had turned out not to be significant. According to Wagenmakers (2007), “assuming the models under consideration are equally plausible a priori, a comparison of their BIC values easily yields an approximation of their posterior probabilities” (p. 796). We found that the BIC approximation of the Bayes factor (BF_01_), expressing the probability of the data given H_0_ (i.e., no interaction) relative to H_1_ (i.e., interaction), was logBF_01_ = 4.8 (for a detailed description of how the BIC approximation of the Bayes factor can be derived see Appendix B in Wagenmakers, 2007, and also Raftery, 1999). Hence, according to the BIC approximation of the Bayes factor (BF_01_), in our experiments H_0_ is logBF = 4.8 times more likely than H_1_. That allows us to accept the null hypothesis according to which the type of mapping in the task performed in the Inducer session did not affect the Simon effect in the Diagnostic session. In other words, the practice effect brought about by the Baseline session (and, possibly, by the Inducer task too) was not affected by the type of mapping employed in the Inducer session.

## Experiment 2

### Method

#### Participants

Sixty-two students of the University of Bologna (43 female, 60 right-handed, *M*_age_ = 22, SD_age_ = 4) served as participants and received course credits for participation. All reported to have normal or corrected-to-normal vision and were naïve as to the purpose of the experiment.

Given *α* (*α* err prob = 0.05), sample size (62), and effect size (*f* = 0.3, Cohen, 1988; see also Verghese, et al,. 2017) the G*power 3.1 software (Faul, et al., 2007) was used to compute post hoc the achieved power to detect a significant interaction between Mapping (Compatible vs. Incompatible), Session (Baseline vs. Diagnostic) and Correspondence (Corresponding vs. Non-corresponding). The power calculation yielded a Power > 99%.

### Apparatus, stimuli and procedure

Apparatus, stimuli and procedure were the same as Experiment 1, with the following exception: participants' eye movements were monitored with the eye tracker system SMI 500 by SensoMotoric Instruments (https://www.smivision.com) at 250 Hz sampling rate. To avoid that an automatic pro-saccade would be triggered by the target stimulus (Kingstone & Praat, 1999; Olk & Kingstone, 2003; see also Lugli, et al., 2016), a distractor stimulus, that is, a non-target stimulus (a green square) was presented simultaneously with the target stimulus (a blue/red square) in the mirror-opposite location. The distractor was a green square (3.05° × 3.05°) and participants were instructed to ignore it.

**Fig. 3:**
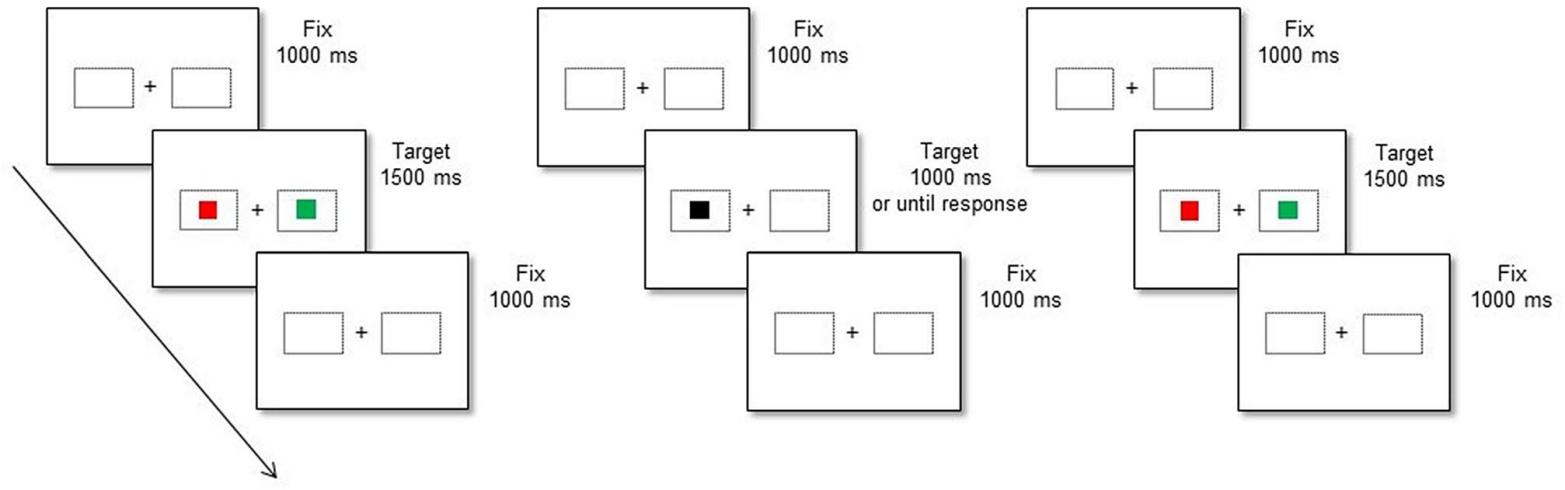
Temporal sequence of a representative trial in the ocular Simon task performed in the Baseline (leftmost panel) and in the Diagnostic ocular Simon task sessions (rightmost panel), and in the spatial compatibility manual task performed in the Inducer session (middle panel). Note that stimuli are not drawn to scale.  See the electornic version for colored figure.

Participants were required to perform first an ocular Simon task (i.e., Baseline session; see Fig. 3, left panel), second a spatial compatibility manual task with a compatible (*N* = 30) or incompatible (*N* = 32) S–R mapping (i.e., Inducer session; see Fig. 3, middle panel) and third an ocular Simon task (i.e., Diagnostic session; see Fig. 3, right panel). In the Baseline and Diagnostic sessions, half of the participants were instructed to direct their gaze toward the left stimulus when the stimulus was red and toward the right stimulus when the stimulus was blue, ignoring the stimulus target location. The other half experienced the opposite mapping rule. At the beginning of the experiment, the fixation cross and the dotted rectangles appeared on the screen and remained visible throughout the whole experiment. Participants were required to fixate the cross, then the stimulus appeared and remained there until the participant had fixated on the stimulus target for at least 1000 consecutive ms.

### Analysis

The latency of initiation of each saccade was computed, that is the Saccadic Reaction Times (ms; SRT) from target stimulus onset to saccade onset. SRTs that were directed to the wrong position (i.e., left instead of right, or vice versa, from the fixation cross) were considered as errors and thus they were not included in the analysis on SRTs (20.1% and 19.1% of the total trials for the Baseline and the Diagnostic sessions, respectively). SRTs that were faster or slower than 2 SD compared to the participant SRT’s mean were excluded (4.7% and 3.5% of the total trials for the Baseline and the Diagnostic sessions, respectively).

The Simon effect, for both the Baseline and the Diagnostic sessions, was computed by comparing Corresponding (i.e., the direction of the saccade corresponded to the position of the stimulus) and Non-corresponding (i.e., the direction of the saccade did not correspond to the position of the stimulus) responses.

As before, eight participants who did not show a positive Simon effect in the Baseline session were excluded from the second analysis, which was thus performed on 54 (39 female, 52 right-handed, *M*_age_ = 22, SD_age_ = 4) out of 62 participants.

Two repeated measures ANOVAs were conducted on both SRTs and arcsine-transformed error rates (ERs) with Mapping (Compatible vs. Incompatible) as between-subject factor and Session (Baseline vs. Diagnostic) and Correspondence (Corresponding vs. Non-corresponding), as within-subjects’ factors, for both samples. Huynh–Feldt correction was used when appropriate. The effect size was estimated by calculating the partial eta squared statistic (*η*_*p*_^2^).

## Results

*SRTs* The first analysis on SRTs on the entire sample (*N* = 62) showed significant main effects of Session *F*_(1, 60)_ = 25.349, MSE = 28,311.606, *p* < 0.001, *η*_*p*_^2^ = 0.297, and Correspondence *F*_(1, 60)_ = 56.115, MSE = 27,322.899, *p* < 0.001, η_p_^2^ = 0.483. Responses were faster in the Diagnostic session (408 ms) than in the Baseline session (430 ms), and in Corresponding (409 ms) than Non-corresponding trials (430 ms).

The interaction Session × Correspondence was significant *F*_(1, 60)_ = 4.939, MSE = 1133.462, *p* = 0.030, *η*_*p*_^2^ = 0.076, indicating that the Simon effect differed between the two sessions, regardless of the Mapping: the Simon effect was of 25 ms in the Baseline session and of 17 ms in the Diagnostic session. Paired sample *t* tests showed that the Simon effects were significant in both sessions (*t*_(61)_ = 7.119, *p* < 0.001 and *t*_(61)_ = 5.204, *p* < 0.001, Baseline and Diagnostic sessions, respectively). See Fig. 2, rightmost panel.

Interestingly, the interaction Mapping × Session × Correspondence was not significant *F*_(1, 60)_ = 0.306, *p* = 0.582, indicating that no modulation occurred between the Simon tasks in the Baseline and Diagnostic sessions related to the Mapping performed in the Inducer session. No other main effects, nor interactions were significant (*F*_*s*_ < 1).

The second analysis on SRTs, on the sample including only participants with an initially positive Simon effect (*N* = 54), showed significant main effects of Session *F*_(1, 52)_ = 18.961, MSE = 20,056.569, *p* < 0.001, *η*_*p*_^2^ = 0.267 and Correspondence *F*_(1, 52)_ = 66.454, MSE = 31,374.464, *p* < 0.001, *η*_*p*_^2^ = 0.561. Responses were faster in the Diagnostic session (406 ms) than in the Baseline session (425 ms), and in Corresponding (403 ms) than Non-corresponding trials (427 ms).

The interaction Session × Correspondence was significant *F*_(1, 52)_ = 12.982, MSE = 2538.123, *p* = 0.001, *η*_*p*_^2^ = 0.200, showing that the size of the Simon effect differed significantly between the two sessions, indicating a reduction of the Simon effect in the Diagnostic session compared to the Baseline session: the Simon effect was of 31 ms in the Baseline session and of 17 ms in the Diagnostic session. Both effects were significant as showed by paired sample t-test (*t*_(53)_ = 8.954, *p* < 0.001 and *t*_(53)_ = 4.785, *p* < 0.001, Baseline and Diagnostic sessions, respectively).

Again, the interaction Mapping × Session × Correspondence was not significant *F*_(1, 52)_ = 0.053, *p* = 0.820, indicating that no modulation occurred between the Simon tasks in the Baseline and Diagnostic sessions related to the Mapping performed in the Inducer session. No other main effect, nor interactions were significant (*F*_*s*_ < 1).

*ERs* The first analysis on ER on the entire sample (*N* = 62) showed a significant main effect of Correspondence *F*_(1, 60)_ = 85.049, MSE = 0.759, *p* < 0.001, *η*_*p*_^2^ = 0.586. Participants made more errors in Non-corresponding (23.76%) compared to Corresponding trials (15.29%). No other main effects nor interaction emerged (*F*_*s*_ < 1).

The second analysis on ER on the sample including only participants with positive Simon effect in the Baseline session (*N* = 54) showed a significant main effect of Correspondence *F*_(1, 52)_ = 86.645, MSE = 0.755, *p* < 0.001, *η*_p_^2^ = 0.625. Participants made more errors in Non-corresponding (24.88%) compared to Corresponding trials (15.78%). No other main effects nor interaction emerged (*F*_*s*_ < 1).

### Bayesian analyses

As described above, this analysis was aimed at comparing the relative plausibility of the null and the alternative hypotheses concerning the three-way interaction involving Mapping, Session and Correspondence, which was not significant. We found that the BIC approximation of the Bayes factor (BF_01_), expressing the probability of the data given H_0_ (i.e., no interaction) relative to H_1_ (i.e., interaction), was logBF_01_ = 7.2 (for a detailed description of how the BIC approximation of the Bayes factor can be derived, see Appendix B in Wagenmakers, 2007, and also Raftery, 1999). Hence, according to the BIC approximation of the Bayes factor (BF_01_), in our experiments H_0_ is logBF = 7.2 times more likely than H_1_. That, again, renders it plausible to accept the null hypothesis according to which the type of mapping performed in the Inducer session did not affect the Simon effect observed in the Diagnostic session.

## Experiment 3^5^

### Method

#### Participants

Twenty-seven students of the University of Bologna (16 females, all right-handed, M_age_ = 20.7, SD_age_ = 3.8) served as participants and received course credits for participation. All reported to have normal or corrected-to-normal vision and were naïve as to the purpose of the experiment.

#### Apparatus, stimuli and procedure

Apparatus, stimuli and procedure were the same as Experiment 1, except that stimuli presentation and response collections were controlled by E-Prime Professional v2.0 software (https://www.pstnet.com). Stimuli were black circle and triangle (3.05° × 3.05°) that appeared at the center of two dotted rectangles (5.05° × 6.5°) presented to the left or to the right of a fixation cross.

Participants were required to perform first a manual Simon task (i.e., Baseline session), second a spatial compatibility manual task with an incompatible S–R mapping (i.e., Inducer session) and third a manual Simon task (i.e., Diagnostic session).

Specifically, in the Inducer session, participants were asked to respond as quickly and accurately as possible to the left stimulus by pressing the right response key and to the right stimulus by pressing the left response key (the “Ctrl” and the “Alt” keys on the QWERTY keyboard, respectively).

In the Baseline and Diagnostic sessions, half of the participants were instructed to respond to the circle stimulus by pressing the right response key and to the triangle stimulus by pressing the left response key (the “Ctrl” and the “Alt” keys on the QWERTY keyboard, respectively). The other half experienced the opposite mapping rule.

### Analysis

Reaction times (RTs) that were 2 SD faster or slower than each participant’s mean (3.80% and 3.28% of the total trials for the Baseline and the Diagnostic sessions, respectively) and errors (2.87% and 4.09% of the total trials for the Baseline and the Diagnostic sessions, respectively) were excluded from the analysis on RTs.

As before, four participants who did not show a positive Simon effect in the Baseline session were excluded from the second analysis, which was thus performed on 23 (12 female, all right-handed, *M*_age_ = 20.5, SD_age_ = 3.8) out of 27 participants.

Two repeated measures ANOVAs were conducted on both RTs and arcsine-transformed error rates (ERs) with Session (Baseline vs. Diagnostic) and Correspondence (Corresponding vs. Non-corresponding), as within-subjects’ factors, for both samples. Huynh–Feldt correction was used when appropriate. The effect size was estimated by calculating the partial eta squared statistic (*η*_p_^2^).

## Results

*RTs* The first analysis on RT on the entire sample (*N* = 27) showed significant a main effects of Session *F*_(1, 26)_ = 12.848, MSE = 205,556.667, *p* = 0.001, *η*_*p*_^2^ = 0.331 and Correspondence *F*_(1, 26)_ = 16.383, MSE = 9203.099, *p* < 0.001, *η*_*p*_^2^ = 0.387. Responses were faster in the Diagnostic session (476 ms) than in the baseline session (503 ms), and in corresponding (480 ms) than non-corresponding trials (499 ms).

The interaction Session × Correspondence failed to reach significance *F*_(1, 26)_ = 3.198, *p* = 0.085 indicating that the Simon effect did not differ between the two sessions: 24 ms in the Baseline session and 13 ms in the Diagnostic session. Both effects were significant as shown by paired sample *t* tests (*t*_(26)_ = 3.853, *p* = 0.001 and *t*_(26)_ = 2.753, *p* = 0.011, Baseline and Diagnostic sessions, respectively).

The second analysis on RTs, performed on the sample with only participants (*N* = 23) with a positive Simon effect in the Baseline session, showed significant main effects of Session *F*_(1, 22)_ = 8.403, MSE = 15,409.947, *p* = 0.008, *η*_*p*_^2^ = 0.276 and Correspondence *F*_(1, 22)_ = 23.593, MSE = 12,094.740, *p* < 0.001, *η*_*p*_^2^ = 0.517. Responses were faster in the Diagnostic session (477 ms) than in the Baseline session (503 ms), and in Corresponding (478 ms) than Non-corresponding trials (501 ms).

The interaction Session × Correspondence was significant *F*_(1, 22)_ = 4.597, *p* = 0.043, *η*_*p*_^2^ = 0.173 indicating that the Simon effect differed between the two sessions: 30 ms in the Baseline session and 15 ms in the Diagnostic session. Both effects were significant, as shown by paired sample *t* tests (*t*_(22)_ = 4.739, *p* < 0.001 and *t*_(22)_ = 2.950, *p* = 0.00, Baseline and Diagnostic sessions, respectively).

*ERs* The first analysis on ER on the entire sample (*N* = 27) showed significant main effects of Session *F*_(1, 26)_ = 24.036, MSE = 0.066, *p* < 0.001, *η*_*p*_^2^ = 0.480 and Correspondence *F*_(1, 26)_ = 12.124, MSE = 0.059, *p* = 0.002, η_p_^2^ = 0.318. Participants made more errors in the Diagnostic session (4.1%) than in the Baseline session (2.9%), and in Non-corresponding (4.1%) compared to Corresponding trials (2.8%). The interaction Session × Correspondence failed to reach significance *F*_(1, 26)_ = 2.913, MSE = 0.0.13, *p* = 0.100. It is worth of note, however, that participants, in both Baseline and Diagnostic sessions, made more errors in Non-corresponding (3.8% and 4.4%, respectively) compared to Corresponding trials (1.9% and 3.7%, respectively). The difference was significant in both sessions (*t*_(26)_ = 3.549, *p* = 0.001 and *t*_(26)_ = 3.432, *p* = 0.002, respectively for the Baseline and the Diagnostic sessions).

The second analysis on ER on the sample including only participants with a positive initial Simon effect (*N* = 23) showed a significant main effects of Session *F*_(1, 22)_ = 19.704, MSE = 0.059, *p* < 0.001, *η*_*p*_^2^ = 0.472 and Correspondence *F*_(1, 22)_ = 16.751, MSE = 0.071, *p* < 0.001, *η*_*p*_^2^ = 0.432. Participants made more errors in the Diagnostic session (4.4%) than in the Baseline session (3.1%), and in Non-corresponding (4.5%) compared to Corresponding trials (3.1%). The interaction Session × Correspondence failed to reach significance *F*_(1, 22)_ = 3.326, MS*E* = 0.017, *p* = 0.082. Again, participants, in both Baseline and Diagnostic sessions, made more errors in Non-corresponding (4.3% and 4.8%, respectively) compared to Corresponding trials (1.9% and 4.0%, respectively). The difference was significant in both sessions (t_(22)_ = 4.103, *p* < 0.001 and *t*_(22)_ = 3.745, *p* = 0.001, respectively for the Baseline session and the Diagnostic session).

## Discussion

We tested the hypothesis that the modulation of the Simon effect performed in the Diagnostic session observed in paradigm that comprises three sessions (i.e., Baseline, Inducer and Diagnostic tasks; see, e.g., Verghese, et al., 2017) is attributable to practice (see Proctor & Lu, 1999) rather than to transfer. We reasoned that if the hypothesis of an effect of practice were correct, the Simon effect observed in the Diagnostic session should decrease in magnitude, regardless of the spatial compatibility mapping and the response modalities performed in the Inducer session. In contrast, if the hypothesis of an effect of transfer were correct, the Simon effect in the Diagnostic session should be affected by the type of mapping employed in the Inducer session. That is, the Simon effect in the Diagnostic session should remain unchanged or increase after a compatible mapping in the Inducer session. In contrast, it should reverse after an incompatible mapping in the Inducer session. We investigated this hypothesis with the same (i.e., manual) response modality in the Inducer and in the Diagnostic sessions, as well as with different response modalities (i.e., manual and ocular) in the two sessions. We employed manual responses in the Inducer session and manual (Experiment 1) or ocular (Experiment 2) responses in the Diagnostic session (as well as in the Baseline session).

Both experiments were comprised of three sessions. These were first the Simon task in the Baseline session (ocular or manual), second the compatibility task in the Inducer session (manual, with a compatible or an incompatible mapping), and third the Simon task in the Diagnostic session (ocular or manual). Note that task requirements, including response modality, were identical in the Simon task performed in both Baseline and Diagnostic sessions. Results showed that, in the three experiments, regardless of the mapping in the Inducer session (i.e., compatible or incompatible) and of the response modality (i.e., same or different), the Simon effect in the Diagnostic session, compared to the Baseline session, was still present but significantly reduced. In our view, the evidence obtained speaks in favor of the fact that, in the Simon task observed in the Diagnostic session, a practice effect rather than a transfer effect occurred, resulting, as predicted by the hypothesis, from the influence of previous tasks performed in the Baseline session and in the Inducer sessions. Very importantly, in the Diagnostic session the Simon effect, though reduced in magnitude, was still present. It did not reverse as should have happened if the hypothesis of a transfer effect (with an incompatible mapping) were correct. However, as was pointed out by an anonymous reviewer, at the present stage, we cannot rule out the alternative possibility that the two effects co-exist.

A possibly critical issue is whether it was correct to exclude the subjects that did not show a positive Simon effect in the Baseline session. By doing so, we excluded those subjects that lacked the effect we wanted to investigate. We opted for the legitimacy of this decision, in accordance with what Verghese, et al. (2017); also see D’Ascenzo, et al. (2016) had done. That is, in accordance with the only study that had tackled the same issue we intended to address.

It must be stressed, however, the importance of the Baseline session in our study. At variance with the majority of previous studies, in which the Baseline session is missing, we in effect asked our subjects to perform the Simon task twice (in the Baseline session and in the Diagnostic session). This way we have submitted our subject to extended practice. Previous studies on transfer effect that included two sessions (i.e., the Inducer and the Diagnostic sessions, without the Baseline session), and implemented a spatially incompatible S-R mapping in the Inducer session, revealed null or reverse Simon effects in the Diagnostic session (e.g., Iani, et al., 2009; Rubichi, et al., 2005; Soetens, et al., 2010). In contrast, when a spatially compatible S-R mapping was implemented, a reduced Simon effect compared to a possibly standard Simon effect, but nonetheless a significant Simon was observed (see, e.g., Iani, et al., 2009, experiment 2). In our study, the Simon effect in the Diagnostic session after performance a task with an incompatible S-R mapping in the Inducer session did not reverse, rather it was still positive and significant. That was true regardless of whether same or different response modalities between Diagnostic and Inducer sessions were employed. In our study, thus, which included three sessions (i.e., Baseline, Inducer and Diagnostic session), the task performed in the Inducer session did affect the Diagnostic session but did not reverse it. In contrast, a reversal occurred in previous studies in which only two sessions were run (i.e., Inducer and Diagnostic sessions).

The reduction in magnitude of the Simon effect we observed in both experiments is comparable to the results obtained by Proctor & Lu, (1999). In their study, after many trials, the Simon effect was still present, although reduced in magnitude. The same reduction was reported by Verghese, et al., (2017), whose main aim was investigating cross response modality transfer effects rather than practice effects. The author implemented a paradigm with three sessions (i.e., Baseline, Inducer and Diagnostic sessions) and concluded that their results were attributable to a transfer effect. However, since no comparisons between the Simon effects in the Diagnostic session after an incompatible task and after a compatible task in the Inducer session were performed, there was no way to ascertain whether what they had found was a transfer effect or a practice effect. In contrast, our results suggest that the transfer effect, at least when investigated with more than two sessions, is likely be overcome by the practice effect.

An issue that deserves to be mentioned concerns still other consequences of running a Baseline session. An advantage of this procedure is that, being the Baseline session performed at the start of the experiment, it allows one to identify participants that have a negative Simon effect, and, thus, should be dropped from the main analyses. This aspect cannot be checked in transfer paradigms with only the Inducer and Diagnostic sessions. Also, the Baseline session is crucial to compare the Simon effect obtained in it with the one obtained in the Diagnostic session. This comparison allows one to estimate the effectiveness of the Inducer session in modulating the Simon effect. However, the Baseline session employed in the present paper can be considered as a double-edged sword, since it can also alter the transfer effect itself, acting as an extra practice that can influence the effect of the Inducer session. It is possible that, by performing the Simon task twice (i.e., in the Baseline and Diagnostic sessions), participants learned to ignore or to suppress the task-irrelevant locational information, resulting in a reduced Simon effect in the Diagnostic session.

For what concerns cross response modalities, we showed that same or different response modalities between the Inducer and both Baseline and Diagnostic sessions did not matter much. The same reduction in the Diagnostic session was observed in either experiment, that is, when same (experiment 1) or different (experiment 2) response modalities were employed. This evidence supports the occurrence of a practice effect rather than of a transfer effect. That is because the former predicts a reduction of the Simon effect in the Diagnostic session, regardless of a difference in the response modalities between the Inducer and the Diagnostic sessions.

In the discussion so far, we have given much weight to the fact that subjects practiced a task in which the relevant feature for both stimulus and the response was spatial in nature. Based on this notion, we assumed that our Baseline session acted by providing the subjects with additional practice with stimulus–response pairs that were spatially defined. If our hypothesis holds, one would predict that a similar effect of practice should be observed in all (rather rare) studies in which a Baseline session was employed. In agreement with the results of the present study, D’Ascenzo, et al., (2016) found that, after a Baseline session, an Inducer session with an incompatible task brought about, in the Diagnostic session, a much decreased Simon effect (from 33 to 6 ms). Admittedly, however, the results of Wang & Weeks (2014) do not fit with the framework we are proposing here. They found that, after an incompatible Inducer session, the Simon effect reversed from 24 ms in the Baseline session to − 10 ms in the Diagnostic session.

In conclusion, the aim of the present study was to disentangle practice effects and transfer effects in a Simon task performed in the Diagnostic session, with the use of a sequential transfer of learning paradigm. Our evidence supports the notion that, in the three-session paradigm, the practice effect prevails over the transfer effect. That is because a reduction of the Simon effect in the Diagnostic session emerged, regardless of the type of mapping, compatible or incompatible, and regardless of the response modality, manual or ocular, that characterized the task performed in the Inducer session. However, the co-existence of the two types of effect cannot be ruled out, and further experiments are necessary to clarify this issue.

## Footnotes

1 In a previous version of the manuscript, in which four experiments were presented by considering each Mapping separately, we calculated the sample size required to achieve 90% power to detect a significant Session (Baseline vs. Diagnostic) × Correspondence (Corresponding vs. Non-corresponding) interaction with the G*power 3.1 (Faul, Erdfelder, Lang, and Buchner, 2007) software. With an effect size f = 0.3 (Cohen 1988; see also Verghese et al. 2017), the power calculation yielded a recommended sample size of at least 22 participants, for each of the four experiments.

2 Note that target stimuli and the distractor stimuli in the Experiments 2 were the same as those used in Lugli, D’Ascenzo, Nicoletti and Umiltà (2017; experiment 2).

3 Note that the same criterion was adopted in the following experiment.

4 The analysis was conducted with the R software program (R Core Team, 2016) using the lme479 library.

5 Based on the recommendations of an anonymous reviewer, Experiment 3 was conducted to explore the possibility that the results of Experiments 1 and 2 were attributable to the dissimilarity between the stimuli employed in the Inducer session versus the Baseline and Diagnostic sessions.
